# Light-independent phospholipid scramblase activity of bacteriorhodopsin from *Halobacterium salinarum*

**DOI:** 10.1038/s41598-017-09835-5

**Published:** 2017-08-25

**Authors:** Alice Verchère, Wei-Lin Ou, Birgit Ploier, Takefumi Morizumi, Michael A. Goren, Peter Bütikofer, Oliver P. Ernst, George Khelashvili, Anant K. Menon

**Affiliations:** 1 Department of Biochemistry, Weill Cornell Medical College, 1300 York Avenue, New York, New York, 10065 USA; 2 Department of Biochemistry, University of Toronto, 1 Kings College Circle, Toronto, Ontario, Canada M5S 1A8; 3 Institute of Biochemistry and Molecular Medicine, University of Bern, 3012 Bern, Switzerland; 4 Department of Molecular Genetics, University of Toronto, 1 Kings College Circle, Toronto, Ontario, Canada M5S 1A8; 5 Department of Physiology and Biophysics, and Institute for Computational Biomedicine, Weill Cornell Medical College, 1300 York Avenue, New York, New York, 10065 USA

## Abstract

The retinylidene protein bacteriorhodopsin (BR) is a heptahelical light-dependent proton pump found in the purple membrane of the archaeon *Halobacterium salinarum*. We now show that when reconstituted into large unilamellar vesicles, purified BR trimers exhibit light-independent lipid scramblase activity, thereby facilitating transbilayer exchange of phospholipids between the leaflets of the vesicle membrane at a rate >10,000 per trimer per second. This activity is comparable to that of recently described scramblases including bovine rhodopsin and fungal TMEM16 proteins. Specificity tests reveal that BR scrambles fluorescent analogues of common phospholipids but does not transport a glycosylated diphosphate isoprenoid lipid. *In silico* analyses suggest that membrane-exposed polar residues in transmembrane helices 1 and 2 of BR may provide the molecular basis for lipid translocation by coordinating the polar head-groups of transiting phospholipids. Consistent with this possibility, extensive coarse-grained molecular dynamics simulations of a BR trimer in an explicit phospholipid membrane revealed water penetration along transmembrane helix 1 with the cooperation of a polar residue (Y147 in transmembrane helix 5) in the adjacent protomer. These results suggest that the lipid translocation pathway may lie at or near the interface of the protomers of a BR trimer.

## Introduction

Phospholipids move rapidly within biological membranes, with in-plane rotational and lateral diffusion occurring on a nanosecond time scale^1^. However, transverse diffusion - the process by which a phospholipid reorients from one side of the bilayer to the other - is very slow, occurring with a frequency of only ~10^−5^ s^−1^ because of the energy barrier that must be overcome in order to transfer the polar head group of the phospholipid through the hydrophobic interior of the membrane^1, 2^. Yet, fast lipid flip-flop is crucial for a variety of cellular processes including i) expansion of the bilayer of biogenic membranes, such as the endoplasmic reticulum (ER), following asymmetric synthesis of phospholipids in the cytoplasmic leaflet^3^, ii) glycosylation pathways such as protein *N*-glycosylation and *O*-mannosylation in the ER^4^ and peptidoglycan synthesis in bacteria^5, 6^, and iii) the maintenance and dissipation of lipid asymmetry at the plasma membrane of eukaryotic cells^1, 7^. In some instances, lipid translocation is carried out by ATP-driven transporters that move lipids against a concentration gradient at a frequency of 10–100 s^−1^. However, many lipid translocation events in cells are not coupled to the consumption of metabolic energy and are facilitated by scramblases, proteins that are presumed to lower the energy barrier for transbilayer movement thereby accelerating bidirectional flip-flop^1^. Scramblase proteins eluded molecular identification until recently when two scramblases were conclusively identified and their transport activities verified by biochemical reconstitution of purified proteins into large unilamellar vesicles^1^. Both scramblases are members of large protein families - Class A G protein-coupled receptors exemplified by the visual pigment rhodopsin^8^, and the TMEM16 family of Ca^2+^-dependent channels/scramblases exemplified by the fungal proteins afTMEM16 and nhTMEM16^9^.

Rhodopsin-like photoreceptor proteins are found in archaea and exhibit some common structural features with mammalian rhodopsin: they have seven transmembrane helices and a retinal chromophore attached in a Schiff base linkage to a lysine residue in the seventh helix^10, 11^. Bacteriorhodopsin (BR) from *Halobacterium salinarum* (also known as *Halobacterium halobium*) is the best studied of this large class of retinylidene proteins^11–13^. BR is expressed in purple membrane patches where it forms trimers that are tightly packed in a highly organized hexagonal lattice^12–16^. Each BR monomer has a covalently attached retinal molecule in the core of the protein. Upon illumination, retinal isomerizes, thereby triggering a cascade of conformational changes that result in the unidirectional active transport of protons against their concentration gradient^12, 13^. In the purple membrane, the light-induced transmembrane electrochemical gradient created by BR is used by adenosine triphosphate (ATP) synthase to produce ATP^12, 13^.

Here we report our finding that BR has robust phospholipid scramblase activity, comparable to that observed for opsin^17, 18^ and Ca^2+^-activated TMEM16 proteins^19–21^. We purified BR as a trimer from n-dodecyl β-D-maltoside (DDM) solubilized purple membrane and reconstituted it into large unilamellar liposomes. The reconstituted protein displayed light-dependent proton pumping activity and scrambled phospholipids in a light-*in*dependent manner at a rate >10,000 s^−1^ per trimer. We hypothesized that a string of surface-exposed polar amino acids in transmembrane helices 1 and 2 of BR may provide a path for phospholipid translocation by coordinating the polar headgroups of transiting phospholipids. Consistent with this proposal, extensive coarse-grained molecular dynamics simulations of a BR trimer in an explicit phospholipid membrane revealed a number of water molecules along transmembrane helix 1, demonstrating the polarity of the proposed pathway. We found that the number of penetrant water molecules depended on the Y147 residue from transmembrane helix 5 of the adjacent protomer suggesting that the lipid translocation pathway may go through the interface of the protomers of a BR trimer.

## Results

**Figure 1:**
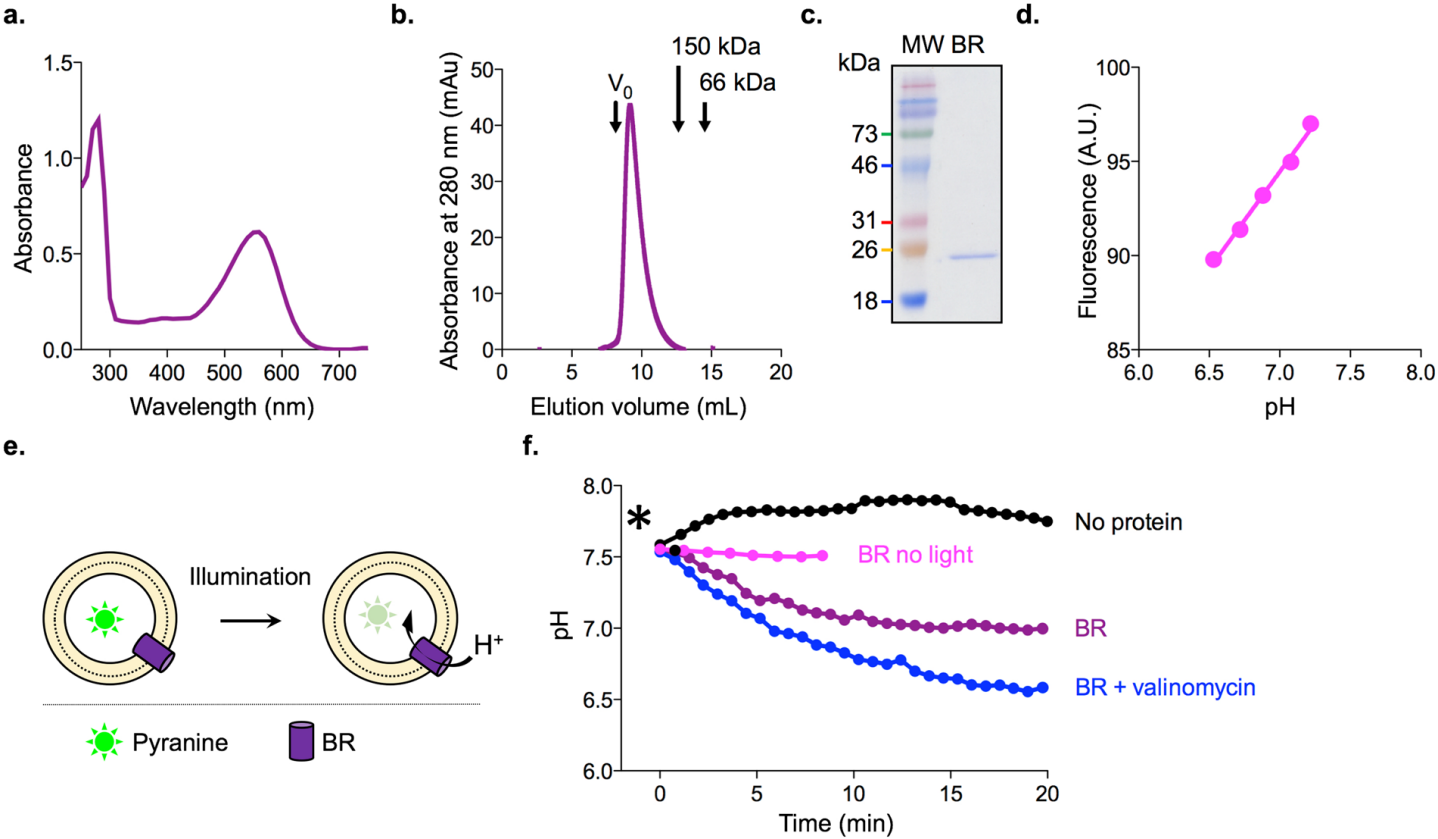
Biochemical characterization of BR purified from purple membrane. (**a**) Absorbance spectrum of purified BR. The ratio of peak heights, A_560_/A_280_ = 0.6/1.22, indicates that the sample is 98% pure. (**b**) Size exclusion chromatography (SEC) purification of BR using a Sephadex 200 column. The void volume V_o_ (determined using Blue Dextran 2,000 kDa) and the elution positions of alcohol dehydrogenase (150 kDa) and bovine serum albumin (66 kDa) are indicated. (**c**) Coomassie-stained SDS-PAGE of purified BR. (**d**) Fluorescence of pyranine as a function of pH (calibration plot). (**e**) Schematic representation of the proton pumping assay. (**f**) pH inside protein-free liposomes (‘no protein’, pH_20_ = 7.57 ± 0.2, mean ± s.d. n = 6) and BR-proteoliposomes (‘BR’, pH_20_ = 6.90 ± 0.14, mean ± s.d. n = 3) upon illumination at time = 0 min (indicated by *). Samples that were either not illuminated or supplemented with valinomycin (final concentration 50 nM, pH_20_ = 6.45 ± 0.16, mean ± range. n = 2) are indicated; pH_20_ corresponds to the pH value 20 min after the start of the experiment.

We purified BR from the purple membrane of *Halobacterium salinarum* using a standard procedure^14^. The purified protein (in DDM) had a characteristic absorbance spectrum with peaks at 280 nm and 560 nm corresponding to the protein and pigment, respectively (Fig. 1a). The ratio of the amplitudes of the two peaks indicates that the preparation is 98% pure (Fig. 1a)^22^. Size exclusion chromatography (SEC) revealed a single monodisperse peak (Fig. 1b) that eluted at a position consistent with trimeric BR in a DDM micelle^23^. Analysis of the SEC-purified material by SDS-PAGE showed a single band at the expected molecular mass of a BR monomer (Fig. 1c). To verify the light-dependent proton pumping activity of our BR preparation we reconstituted the protein into liposomes containing pyranine, a fluorophore whose fluorescence is pH-dependent^24^ (Fig. 1d). As illustrated schematically in Fig. 1e, illumination of BR is expected to result in protons being transported to the interior of the liposomes^25^, leading to a drop in pH and a corresponding decrease of pyranine fluorescence. Because the decrease in pyranine fluorescence is linearly proportional to the pH inside the liposome at the pH range of this assay^24^ (Fig. 1d), we could deduce the pH inside the liposomes. As expected we found light-dependent acidification of liposomes containing BR, with no acidification seen in vesicles that lack the protein (Fig. 1f). Both the rate and extent of acidification were increased on eliminating the transmembrane gradient of positive charge with the K^+^ ionophore valinomycin (Fig. 1f). We conclude that our BR preparation consists of properly folded trimeric BR with light-dependent proton-pumping activity.

**Figure 2:**
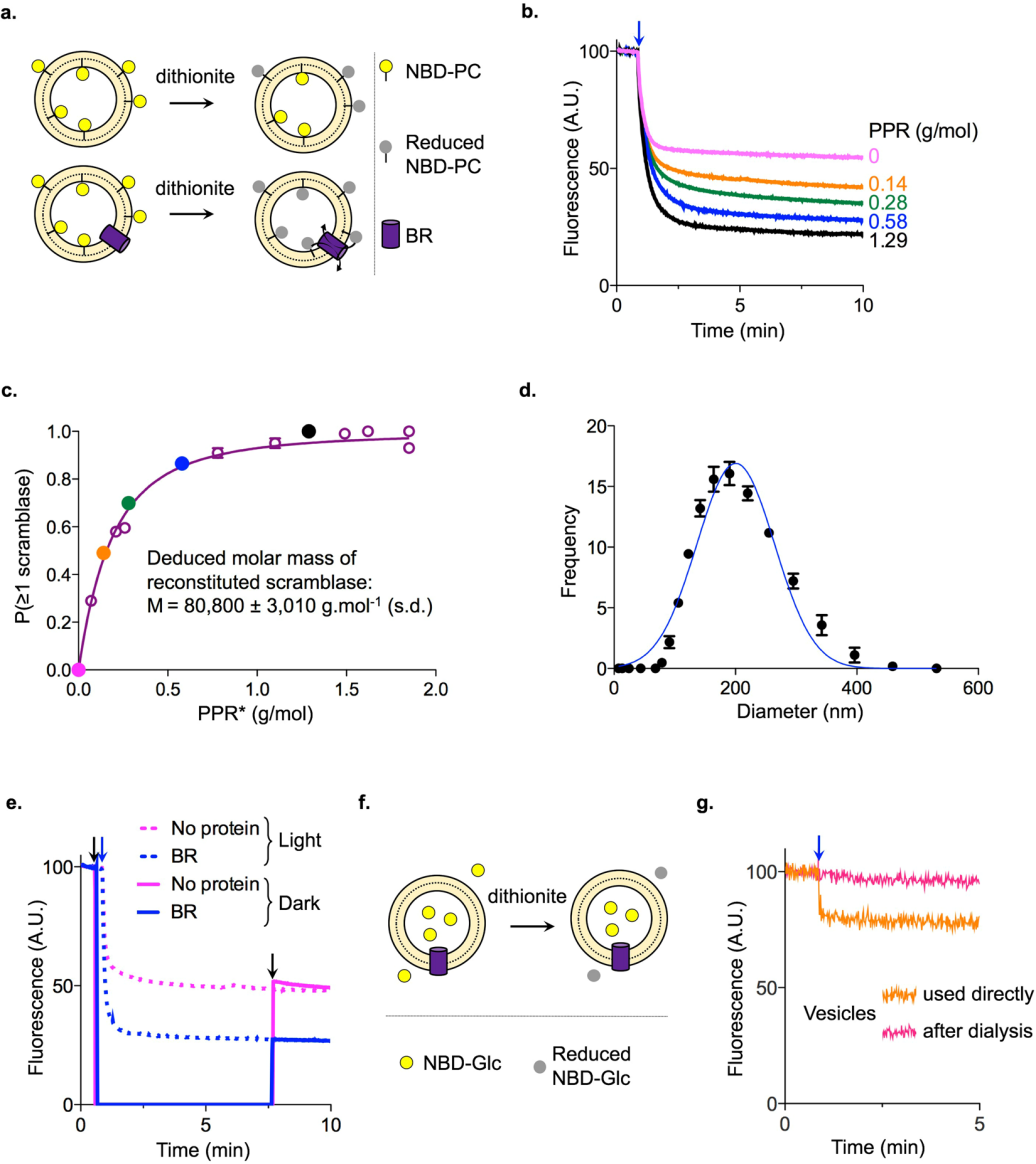
Scramblase activity of BR. (**a**) Schematic representation of the scramblase assay. (**b**) Fluorescence traces from a scramblase assay using NBD-PC as the reporter lipid. Dithionite addition (blue arrow) leads to a 50% decrease of fluorescence for protein free liposomes (pink trace) and a greater reduction for BR-containing proteoliposomes, the extent of which depends on the protein to phospholipid ratio (PPR). (**c**) Probability that a vesicle has at least one functional scramblase (*P* (≥*1 scramblase*)) as a function of PPR* (PPR* is obtained from the measured PPR (Methods) and data fitting was done according to equation (2)). Solid coloured data points correspond to the traces shown in panel b. (**d**) Size distribution of liposomes measured by dynamic light scattering (each point is the mean ± s.d. of a triplicate analysis). The data were analyzed using a Gaussian distribution (line): average radius of the vesicles $\bar{{\rm{r}}}$ = 100.6 nm, standard deviation σ=31.5 nm. (**e**) Scramblase assay in the light versus dark. The plot shows NBD fluorescence as a function of time. Traces in dotted lines correspond to liposomes (protein free) and BR proteoliposomes during a normal scramblase assay where the sample is continuously illuminated by NBD excitation light in the cuvette; solid traces correspond to the assay in the dark performed as follows after briefly recording fluorescence of the sample: 1^st^ arrow: cuvettes are placed in the dark, 2^nd^ arrow: dithionite is added (also in the dark), with continuous stirring, 3^rd^ arrow: cuvettes are placed back into the fluorimeter for fluorescence measurement. (**f**) Schematic representation of the assay to test dithionite permeation into liposomes. (**g**) NBD-glucose was encapsulated in liposomes during reconstitution and the vesicles were used directly (orange) or after dialysis to remove extravesicular NBD-Glucose (pink). Fluorescence was recorded continuously and dithionite was added as indicated (blue arrow). For vesicles used directly after reconstitution, dithionite addition caused a sharp drop in fluorescence followed by a steady signal (orange trace). This corresponds to reduction of extravesicular NBD-Glucose and protection of the encapsulated pool. The extent of reduction was not as great as expected based on the ratio of extravesicular versus intravesicular volume because of significant adsorption of NBD-Glucose to the BioBeads. For the dialyzed preparation, dithionite addition caused no change in fluorescence (pink trace).

We next used a well-established assay^17, 26–28^ to determine whether BR can scramble phospholipids across a membrane bilayer. In this assay (Fig. 2a), the protein is reconstituted into liposomes containing a trace amount of a fluorescent reporter lipid (1-palmitoyl-2-{6-[7-nitro-2–1,3-benzoxadiazol-4-yl)amino]hexanoyl}-*sn*-glycero-3-phospho-choline; NBD-PC). Upon reconstitution, NBD-PC is equally distributed between the two leaflets of the liposome with one half of all NBD-PC molecules in the inner leaflet and the other half in the outer leaflet^17, 27^. When protein-free liposomes are treated with dithionite, a membrane-impermeant reducing agent^18, 29^, the NBD fluorophore is irreversibly reduced and thereby rendered non-fluorescent. Thus, adding dithionite to protein-free liposomes is expected to result in a ~50% reduction in fluorescence, because only the NBD lipids in the outer leaflet are reduced (Fig. 2a, upper panel). When scramblase-containing proteoliposomes are analyzed, the fluorescence decrease is expected to be 100% as phospholipids are flip-flopped across the membrane enabling NBD-PC in the inner leaflet to reach the dithionite accessible outer leaflet (Fig. 2a, lower panel).

We reconstituted BR using a standard procedure^28, 30^ in which preformed large unilamellar liposomes composed of a 9:1 mixture of phosphatidylcholine (POPC) and phosphatidylglycerol (POPG) were destabilized with DDM, combined with DDM-solubilized BR and NBD-PC, and treated with detergent-adsorbing BioBeads to enable reconstitution of the protein. Addition of dithionite to protein-free liposomes resulted in ~50% reduction of NBD-PC fluorescence, whereas the extent of reduction was >50% for BR-containing liposomes (Fig. 2b), indicating scrambling of lipids from the inner to the outer leaflet. The extent of reduction depended on the amount of BR used for reconstitution (Fig. 2b), but the rate of fluorescence loss in all BR samples was similar to that seen for protein-free liposomes. Consistent with previous reports of the phospholipid scramblase activity of opsin^17, 18, 31^ and fungal TMEM16 proteins^19–21^, these data indicate that BR is a phospholipid scramblase with high activity such that NBD lipids are scrambled faster than the rate at which the fluorophore is reduced by dithionite. Based on the size of the reconstituted vesicles (each vesicle contains about 3.5 × 10^5^ phospholipids^32^) and the rate constant for reduction (half-life of fluorescence reduction is 15.2 ± 2.8 s, mean ± s.d., n = 17) which provides a lower limit of the BR-facilitated scrambling rate, we estimate that BR scrambles phospholipids at a rate >10^4^ lipids per second.

The extent of fluorescence reduction is a measure of the fraction of vesicles that contain a functional scramblase^17, 18, 31^, and can be used to calculate the probability (*P* (>*1 scramblase)*) that a particular vesicle is equipped with at least one functional scramblase. To do this, the fluorescence data are transformed using the following equation:

1 $$P(\ge 1\,scramblase)=\frac{(F-{F}_{0})}{({F}_{max}-{F}_{0})}$$

where *F*_0_ is the relative fluorescence reduction observed on dithionite treatment of protein-free liposomes (43.0 ± 2.9%, mean ± s.d., n = 4), *F*_*max*_ is the maximal relative fluorescence reduction observed (76.2 ± 1.6%, mean ± s.d., n = 5) for high protein to phospholipid ratio (PPR) values (above 0.8 g BR per mole phospholipid) where all vesicles are expected to be reconstituted with a functional scramblase, and *F* is the relative fluorescence reduction at a particular PPR^18, 19, 31^. Consistent with numerous previous reports^18, 19, 31, 33^, *F*_*max*_ never exceeded ~80% in our experiments, short of the 100% expected value if all vesicles contain a scramblase. This shortfall suggests that a significant fraction of vesicles is refractory to reconstitution possibly because the stochastic nature of detergent removal produces a fraction of vesicles from which detergent has been removed early during treatment, preventing subsequent insertion of protein.

*P*(≥*1 scramblase*) depends on the amount of protein reconstituted, i.e. the PPR of the sample. If we stipulate that a single reconstitution event confers scramblase activity to that vesicle, then according to the Poisson law *P*(≥*1 scramblase*) should increase mono-exponentially with PPR^17, 18^ (Fig. 2c, but see below). As described previously^31^ the relationship between *P*(≥*1 scramblase*) and PPR becomes more complex when taking into consideration the size distribution of the liposomes (measured by dynamic light scattering before reconstitution, Fig. 2d) and also the fraction of vesicles in which no protein is reconstituted irrespective of the amount of protein used in the reconstitution. We analyzed the data using this improved analytical model (Fig. 2c). From the associated fit constant we calculated the molecular mass of the functional scramblase as 80,800 ± 3,010 g/mol (value ± uncertainty), which corresponds to a BR trimer.

Because BR pumps protons in a light-dependent fashion we tested whether light also affects its scramblase activity. We performed the scramblase assay in the fluorimeter under continuous illumination, or in the dark. For the latter measurement, exciting light was turned on briefly to quantify NBD fluorescence prior to dithionite addition and then again >5 min after dithionite addition to measure the extent of reduction. The extent of fluorescence reduction in the two conditions was identical (Fig. 2e) indicating that BR’s scramblase activity is light-independent.

Although dithionite is well-established as a membrane-impermeant reagent for synthetic vesicle systems^18, 19, 26, 27, 29^, we wanted to ensure that this was indeed the case under our assay conditions. For this we carried out three control experiments. *First*, we encapsulated NBD-Glucose (NBD-Glc) in the vesicles during reconstitution (Fig. 2f). We used a large amount of BR (PPR 1.3 g/mol) to maximize the probability of vesicles containing at least one BR trimer. The vesicles were used immediately after reconstitution or after dialysis to remove extravesicular NBD-Glc. As shown in Fig. 2g, addition of dithionite to vesicles taken directly after reconstitution resulted in a sharp drop in fluorescence corresponding to NBD-Glc present in the extravesicular medium, after which fluorescence intensity remained constant (orange trace, Fig. 2g). Correspondingly, addition of dithionite to dialyzed vesicles (pink trace, Fig. 2g) caused no change in NBD fluorescence. These data indicate that the vesicles are able to protect encapsulated NBD-Glc from dithionite reduction, i.e. dithionite cannot cross the vesicle membrane. *Second*, we noticed that exposure of BR to octyl-β-D-glucoside (β-OG) does not affect its ability to pump protons (Supplementary Fig. S1a), but causes loss of its scramblase activity (Supplementary Fig. S1b). Scramblase activity assays performed on reconstituted, β-OG-treated BR yielded reductions of only ~50% similar to the level observed for protein-free liposomes, even though we used far greater amounts of BR for reconstitution than would be used in a standard assay. This result shows that proteoliposomes reconstituted with ‘scramblase-dead’ BR are able to maintain a protected pool of NBD-PC in the inner leaflet, and are therefore not leaky to dithionite. *Third*, we used a different scramblase assay protocol in which NBD-PC in the outer leaflet is probed by extraction with fatty acid-free bovine serum albumin (BSA) rather than by dithionite reduction. The results of this assay (Supplementary Fig. S2), carried out in parallel with the dithionite reduction method, confirm that BR is a phospholipid scramblase.

We next tested BR’s substrate specificity using several commercially available NBD-lipids, and found that it transports both *N*-NBD-phosphatidylethanolamine (*N*-NBD-PE) and NBD-sphingomyelin (C_12_-NBD-SM) (Supplementary Fig. S3). In contrast with NBD-PC (used in the assays described above) which has a short C_6_-NBD chain esterified to the 2-position of glycerol, *N*-NBD-PE has a more natural diacylglycerol anchor and C_12_-NBD-SM has an NBD-labeled ceramide anchor. Thus, BR is relatively unspecific with respect to its phospholipid substrates. To evaluate BR’s substrate specificity further we next tested Man_5_GlcNAc_2_-PP-dolichol (M5-DLO, an intermediate in the dolichol pathway of protein *N*-glycosylation)^4, 17^ as a potential substrate. This lipid has an extremely long isoprenoid lipid anchor connected via a diphosphate bridge to a heptasaccharide, and we previously showed that it is not scrambled by bovine opsin^17^. Scrambling of M5-DLO was assayed using the mannose-binding lectin Concanavalin A (Con A) as a topological probe (Supplementary Fig. S4). Briefly, proteoliposomes were prepared with a trace amount of [^3^H]-M5-DLO and incubated with Con A. For both protein-free liposomes and BR proteoliposomes prepared at a high PPR~2 g/mol, 47.7% (n = 2) and 47.9% (n = 2) respectively of [^3^H]-M5-DLO was captured by Con A, whereas 87.7% (n = 2) was captured when proteoliposomes were prepared with a crude detergent extract of yeast endoplasmic reticulum membranes containing M5-DLO scramblase^34^ (Supplementary Fig. S4). Thus, like bovine opsin, BR does not scramble M5-DLO.

## Discussion

**Figure 3:**
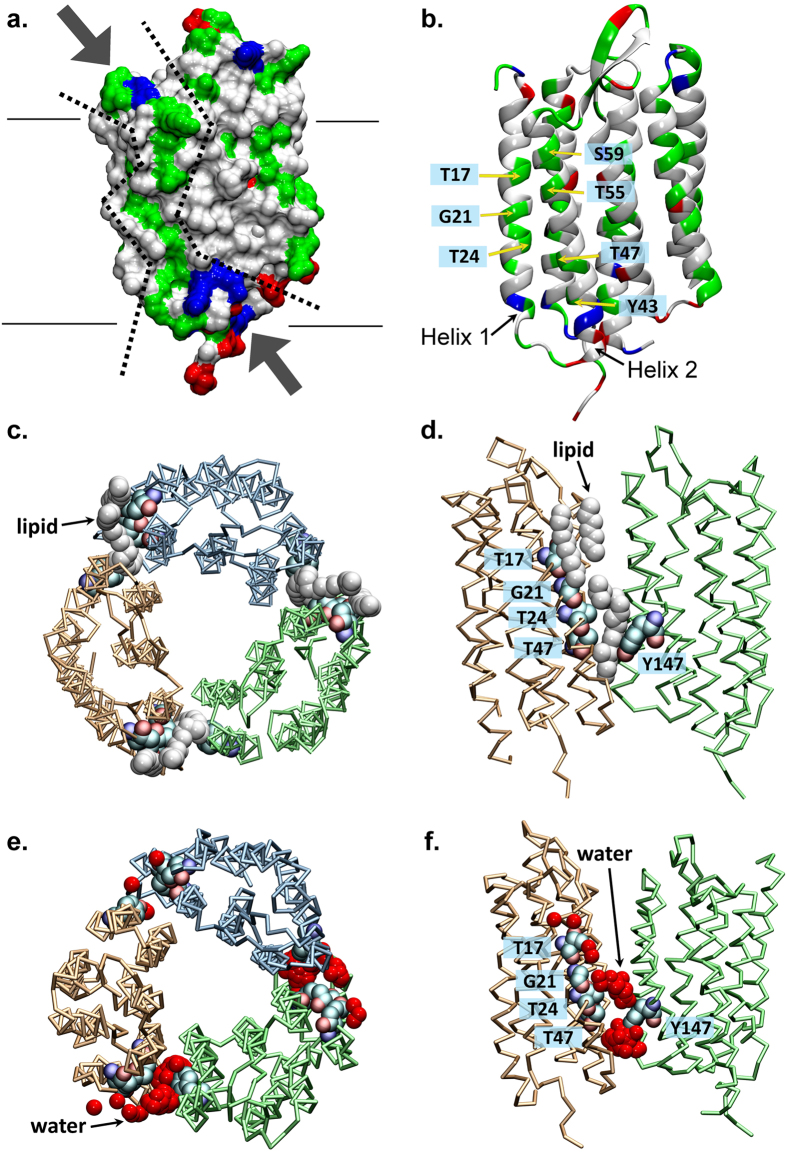
Possible structural basis for phospholipid scrambling by BR. (**a**) Space-fill representation of BR structure (from PDBID: 4MD2), showing positively charged residues (blue), negatively charged residues (red) and polar residues (green). The potential lipid-translocation path is shown within the dotted lines. (**b**) Cartoon model of BR (PDBID: 4MD2) using the same color code as for panel a, specifying the polar amino acid residues that are proposed to form the lipid translocation pathway. (**c**,**d**) Structure of a trimer of BR viewed from the exoplasmic side (**c**) and from the membrane (**d**), generated by the PDBePISA algorithm from PDBID: 4MD2. The residues that are proposed to be involved in the lipid translocation pathway are represented in space fill (and labeled in panel d) and lipid molecules from the structure are drawn in white. The individual BR monomers in the trimer are shown in trace representation and are colored differently – orange, cyan and green; for clarity only 2 protomers of the trimer are depicted in panel d. (**e,f**) Top (**e**) and side (**f**) views of BR (illustrated in trace representation of its backbone) from the CGMD simulations showing accumulation of water molecules near TMs 1 and 2. Different protomers of the trimer are colored as in panels c,d, with relevant residues shown in space fill representation and labeled in panel f. Red spheres represent superposition from all the trajectory frames of Martini water beads (1 bead equivalent to 4 water molecules) within 7 Å of residues T17, G21, T24, and T47. In panel f, only 2 protomers of the trimer are depicted for clarity. Note the involvement of Y147 from the TM5 helix of the adjacent protomer in solvation of the interfacial region.

We report that BR has robust phospholipid scramblase activity, comparable to that of recently described GPCR and TMEM16 family scramblases. Our observation raises two questions: how does BR scramble phospholipids, and what is the physiological relevance of this activity. A popular model to explain scramblase activity postulates that a scramblase operates like a credit card reader, providing a protected transverse pathway (the slot in the card reader) for the transiting phospholipid headgroup (magnetic stripe on the credit card)^1, 35^. Indeed, TMEM16 scramblases may operate by this mechanism as, evinced by the structure of nhTMEM16, they have a membrane-spanning hydrophilic groove that could provide the ‘card-reader’ function^21^. We inspected X-ray structures of BR to identify features that might play a role in lipid scrambling. Interestingly, we found a series of membrane-exposed polar residues in transmembrane helices 1 and 2 (Fig. 3a,b) that provide an almost complete polar path on the surface of the protein, normal to the plane of the membrane, along which lipids could move (residues in green, within dotted lines, Fig. 3a). While these residues are close to the surface of the protein that is involved in trimer formation, they are nevertheless significantly exposed to the membrane (Fig. 3c,d). The structure of BR that we used for this analysis (PDBID: 4MD2) was acquired from lipidic cubic phase crystallization^36^ and reveals a number of phospholipids closely aligned with the polar path (lipids are in white in Fig. 3c,d). While the suggestive locations of these lipid molecules may simply be a result of the crystallization method^37^, they nevertheless indicate that lipids can be accommodated along this surface of the protein, even when BR is organized into trimers (Fig. 3c,d).

In order to test whether the polar path can recruit waters and thus create a conduit for lipid translocation, we performed extensive (~25 μs) Martini molecular dynamics (MD) simulations of a BR trimer in an explicit phospholipid membrane with the same composition as used in our scramblase assays (see Methods). We observed a water path along transmembrane (TM) helix 1 of the protein, in two out of three protomers of the trimer (Fig. 3e,f; water molecules are shown in red) and quantified that as many as 8 water molecules can be simultaneously coordinated by residues T17, G21, T24, and T47, lining the hydrophilic pathway seen in the X-ray structure. Interestingly, we found that the level of solvation around TMs 1 and 2 was affected by polar residues from the adjacent protomer. Thus, residue Y147 (in TM5 of the adjacent protomer) appears to coordinate incoming water molecules before they diffuse towards the polar residues in TMs 1 and 2 (Fig. 3f). Interestingly, our similarly long Martini MD simulations of monomeric BR (taken from the PDBID: 4MD2 structure) in the same membrane and carried out under the same conditions as the trimer, did not show waters along any of the transmembrane segments of the protein, including the interface of TM helices 1 and 2. Thus the polar path for lipid translocation depicted in Fig. 3a may be mechanistically supported by structural contributions from the interface of protomers in the BR trimer. A direct test of this hypothesis is the subject of future work.

The biological role of phospholipid scrambling by BR within the purple membrane patches of *Halobacterium salinarum* is not clear. Purple membrane lipids are typically ether lipids, the most common being phosphatidylglycerol phosphate methyl ester^38, 39^, a lipid that would likely be scrambled given BR’s relaxed phospholipid substrate specificity (Supplementary Figure S3). However, as the purple membrane is in a quasi-crystalline state *in vivo* and since this organization is pivotal for the physiology of BR^40^, it seems unlikely that lipids would be subject to scrambling in this context. Instead, it is interesting to consider that prior to inclusion in the purple membrane the BR polypeptide is co-translationally inserted into the cytoplasmic membrane of the archaeon^38, 41^ where it may function as a scramblase, enabling lipids that are synthesized on the cytoplasmic side of the membrane to populate the exoplasmic leaflet as part of the process of bilayer assembly. Regardless, we speculate that BR or BR-like proteins must have functioned as scramblases in an evolutionary significant setting because the ability to scramble lipids requires very specific structural features. Indeed, membrane proteins in general are not scramblases. A couple of examples serve to highlight this point. *First*, homologous members of the TMEM16 family are distinct with respect to scramblase activity: whereas TMEM16A has no scramblase activity (it is a Ca^2+^-dependent Cl^−^ channel)^19^, TMEM16F and fungal TMEMs (afTMEM16, nhTMEM16) are scramblases^19–21, 42^. Indeed, transplantation of specific sequences from TMEM16F into TMEM16A results in a TMEM16A protein that can scramble phospholipids^42^. Furthermore, elimination of scramblase activity by point mutagenesis of TMEM16 proteins also indicates that scrambling has a highly specific structural basis^43^. *Second*, most membrane proteins of the endoplasmic reticulum (ER) lack scramblase activity as evinced by velocity gradient centrifugation of detergent extracts of rat liver ER or yeast ER. In these experiments, scramblase activity was found in a protein fraction that sedimented at 4S, whereas the great majority of ER proteins ranging though all gradient fractions, lacked activity^3, 27, 44^. Thus, we hypothesize that BR’s specific ability to scramble lipids derives from unique features of its structure (Fig. 3) that may be associated with an ancestral requirement for lipid scrambling. For example, as discussed above, a BR-like protein may have played a role in membrane biogenesis, allowing a biogenic membrane bilayer to expand uniformly in the face of asymmetric synthesis of phospholipids on the cytoplasmic side^45, 46^.

BR was the first polytopic membrane protein to be structurally defined^47^ and it continues to be important as both a model for investigations of membrane protein structure and biogenesis, and as a tool in membrane biology. Indeed, the ready availability and robust physical properties of BR enabled its use as a reporter in the development of numerous biochemical techniques, including the use of BioBeads for membrane protein reconstitution in liposomes^48, 49^, investigation of the properties of amphipols in membrane protein refolding^50^, lipid cubic phase crystallization^51^, detergent-free crystallization^52, 53^, and time-resolved serial femtosecond crystallography^54^. Our discovery of BR’s scramblase activity will likely open the way to new applications.

## Materials and Methods

### Materials

All lipids (1-palmitoyl-2-oleoyl-*sn*-glycero-3-phosphocholine (POPC), 1-palmitoyl-2-oleoyl-*sn*-glycero-3-phospho-(1′-*rac*-glycerol) (POPG), 1-palmitoyl-2-{6-[(7-nitro-2–1,3-benzoxadiazol-4-yl)amino]hexanoyl}-*sn*-glycero-3-phosphocholine (NBD-PC), 1,2-dipalmitoyl-*sn*-glycero-3-phosphoethanolamine-N-(7-nitro-2-1,3-benzoxadiazol-4-yl) (ammonium salt) (*N*-NBD-PE) and N-[12-[(7-nitro-2-1,3-benzoxadiazol-4-yl)amino]dodecanoyl]-sphingosine-1-phosphocholine (NBD-SM) 1-myristoyl-2-{6-[(7-nitro-2-1,3-benzoxadiazol-4-yl)amino]hexanoyl}-*sn*-glycero-3-phosphocholine (myr-NBD-PC)) were purchased from Avanti polar lipids, n-Dodecyl-β-D-Maltopyranoside (DDM) was from Anatrace, BioBeads SM2 adsorbent was from BioRad and NBD-Glucose was from Invitrogen.

### Methods

#### Expression and purification of BR

BR was obtained as previously described^14^. Briefly, *Halobacterium salinarum* cells were grown in a liquid medium containing 4 M NaCl, 150 mM MgSO_4_, 10 mM trisodium citrate, 30 mM KCl, 5 g/L yeast extract, and 5 g/L peptone. Overexpression of BR was induced by illumination of the culture for 7 days at 37 °C. Cells were lysed by osmotic shock in distilled water and purple membrane was isolated by sequential centrifugation, purified using a sucrose gradient and solubilized as follows: a membrane suspension containing 15–20 mg BR at 7 g/L was sonicated for 5 min and incubated for 48–72 h at 4 °C with 2% (w/v) DDM. Non-solubilized material was removed by ultracentrifugation. BR was purified on a Sephadex 200 column (GE Healthcare). The concentration of solubilized proteins was estimated from their extinction coefficient, using ε_560nm_ = 54,000 M^−1^.cm^−1^ and ε_280nm_ = 66,000 M^−1^.cm^−1^ ^55^.

#### Preparation of unilamellar vesicles

Liposome preparation and protein reconstitution were performed as described previously^28^. Briefly, POPC and POPG, solubilized in chloroform, were mixed at a 9:1 molar ratio and dried under vacuum. The resulting lipid film was resuspended in 50 mM HEPES pH 7.4, 100 mM NaCl (2 mM pyranine was added at this step for the proton pumping assay) and extruded through 400 nm and 200 nm membranes. Liposomes were destabilized with DDM at a detergent to lipid ratio of 3 (mol/mol). NBD-PC (0.3% mole percent; palmitoyl-NBD-PC was used for the dithionite assay, and myristoyl-NBD-PC for the fatty acid free BSA assay) and protein in DDM were added before removing the detergent in stages using 3 successive additions of BioBeads. For the proton pumping assay, NBD-PC was omitted and non-encapsulated pyranine was removed using a desalting column (PD10, GE Healthcare). To test dithionite permeation across the vesicle membrane, NBD-Glucose was used instead of NBD-PC; samples were used directly after BioBeads treatment or after removing the non-encapsulated NBD-Glucose by dialysis against the same buffer, using a dialysis membrane with a cut off 8–10 kDa. Lipid content of the liposomes was determined using a colorimetric assay^28, 56^ calibrated against inorganic phosphate standards. The protein content in proteoliposomes was determined by quantitative colloidal blue staining (Invitrogen) of SDS-PAGE for BR compared to purified starting material that was quantified by absorbance at 560 nm using an extinction coefficient of 54,000 M^−1^.cm^−1^. The size distribution of the liposome preparation was measured by dynamic light scattering (Malvern Instruments Ltd, United Kingdom).

#### Fluorescence measurements

All fluorescence measurements were performed at room temperature under constant stirring using a Photon Technology International Inc. fluorescence spectrometer. The excitation and emission slit widths were 0.5 nm.

Proton pumping assay: liposomes were incubated in the dark for at least 20 min before starting the measurement. Pyranine fluorescence (excitation/emission; 455/509 nm) was recorded as a function of time for 20 min with 20 sec illumination pulse/20 sec pause cycles.

Scramblase assay: scramblase activity was measured as described previously^17, 28^. Briefly 50 µL of NBD-PC-containing liposomes were diluted in 1.95  mL of 50 mM HEPES pH 7.4, 100 mM NaCl, and NBD fluorescence (excitation/emission; 470/530 nm) was monitored as a function of time. After 50 s 40 µL of 1 M sodium dithionite (freshly prepared in unbuffered 0.5 M Tris) or 40 µL of fatty acid free BSA at 75 mg/mL was added to the cuvette and fluorescence was recorded for a further 600 s.

#### Analysis of scramblase reconstitution

The analysis was performed as described previously^31^. The rationale of the analysis relies on determining the probability of having at least one scramblase per vesicle *P*(>*1 scramblase*). This probability follows Poisson statistics and since the vesicles have a Gaussian size distribution with a mean radius of $\bar{{\rm{r}}}$ (100.8 nm) and a standard deviation of σ (32.8 nm), the probability can be expressed as follows:

2 $$P(\ge 1\,scramblase)=1-\frac{1}{\sqrt{1+{{\rm{\sigma }}}^{2}{\rm{\alpha }}PP{R}^{\ast }}}{{\rm{e}}}^{\frac{{\bar{{\rm{r}}}}^{2}PP{R}^{\ast }\frac{{\rm{\alpha }}}{2}}{1+{{\rm{\sigma }}}^{2}PP{R}^{\ast }{\rm{\alpha }}}}$$

where α is the fit constant, inversely proportional to the molecular mass of the functional reconstituted scramblase and PPR* = PPR/0.524 is the PPR corrected for the pool of vesicles that is refractory to reconstitution.

Because the functional test for scrambling is an end point assay, we can estimate the value of the probability that a particular vesicle has a functional scramblase using equation (1). When the data set of *P*(≥*1 scramblase*) is plotted versus PPR* and analyzed via equation (2), the fit constant α = 8.96E-04 ± 3.34E-05 mol^−1^.g^−1^.nm^−1^ (value ± uncertainty) corresponds to reconstitution of a functional scramblase with a molecular weight of 80,800 ± 3,010 g.mol^−1^ (value ± uncertainty), i.e. a trimer of BR.

#### Computational Methods

Coarse-grained (CG) molecular dynamics (MD) simulations with Martini force-fields^57, 58^ were performed on a trimer model of BR from PDBID: 4MD2^59^. The spatial arrangement of this structure in a lipid bilayer was first optimized using Orientations of Proteins in Membranes (OPM) database^60^ and then inputted into the Martini Bilayer Maker available on the CHARMM-GUI web-server^61^ in order to assemble a protein-membrane system with Martini CG representations. The Martinized BR model was embedded into a 500-lipid size membrane containing a 9:1 mixture of POPC and POPG lipids. The protein-membrane complex was then solvated and ionized with 0.15 M KCl to achieve electroneutrality.

The assembled CG system was initially minimized and equilibrated following the multi-step protocol provided by CHARMM-GUI and then simulated for ~25 μs (taking into account a factor of 4 speed-up for Martini simulations^58^) in an NPT ensemble using semi-isotropic pressure coupling. Constant pressure was maintained with the Berendsen algorithm^62^. The simulations were implemented a 20 fs integration time-step and run with the elnedyn (Elastic Network in Dynamics) force-field^63^. All the simulations were performed using Gromacs version 5.0.4^64^.

## Electronic supplementary material

Supplementary Material
