# Supplementary material for: Light-independent phospholipid scramblase activity of bacteriorhodopsin from Halobacterium salinarum
Source: Sci Rep. 2017 Aug 25;7:9522. doi: 10.1038/s41598-017-09835-5 (PMC5572738; doi:10.1038/s41598-017-09835-5)
Supplement: Supplementary file 1 — Supplementary Material [file 41598_2017_9835_MOESM1_ESM.pdf]

Supplementary Information for:

Light-independent phospholipid scramblase activity of bacteriorhodopsin from *Halobacterium salinarum*

Alice Verchère[1], Wei-Lin Ou[2], Birgit Ploier[1], Takefumi Morizumi[2], Michael A. Goren[1], Peter Bütikofer[3], Oliver P. Ernst[2,4], George Khelashvili[5], Anant K. Menon[1*]

[1] Department of Biochemistry, Weill Cornell Medical College, 1300 York Avenue, New York, New York, 10065, USA.
[2] Department of Biochemistry, University of Toronto, 1 Kings College Circle, Toronto, Ontario, Canada, M5S 1A8.
[3] Institute of Biochemistry and Molecular Medicine, University of Bern, 3012 Bern, Switzerland.
[4] Department of Molecular Genetics, University of Toronto, 1 Kings College Circle, Toronto, Ontario, Canada, M5S 1A8.
[5] Department of Physiology and Biophysics, and Institute for Computational Biomedicine, Weill Cornell Medical College, 1300 York Avenue, New York, New York, 10065, USA.

*Correspondence:
Anant K. Menon (akm2003@med.cornell.edu)

Exposure of BR to octyl-β-D-glucoside (β-OG) eliminates its scramblase activity without affecting its proton pumping activity.

We solubilized BR from purple membrane at 4ºC using octyl-β-D-glucoside (β-OG). The protein was subsequently incubated for 1 h at room temperature in 20 mM β-OG prior to reconstitution (via β-OG-mediated destabilization of liposomes). This procedure retained BR's ability to pump protons in response to light (Fig. S1a). However, the same protocol resulted in the loss of BR's scramblase activity (Fig. S1b). No scrambling was seen even if the amount of β-OG-treated BR that was reconstituted exceeded the amount normally used in scramblase assays by more than two orders of magnitude. In these BR-containing proteoliposomes, the loss in NBD-PC fluorescence observed on dithionite addition was ~50%, corresponding to chemical reduction of the pool of NBD-lipids in the outer leaflet. While the reason for selective loss of scramblase activity is presently not clear, the presence of a protected pool of NBD-PC indicates that *dithionite cannot access the inner leaflet of BR-containing proteoliposomes*.

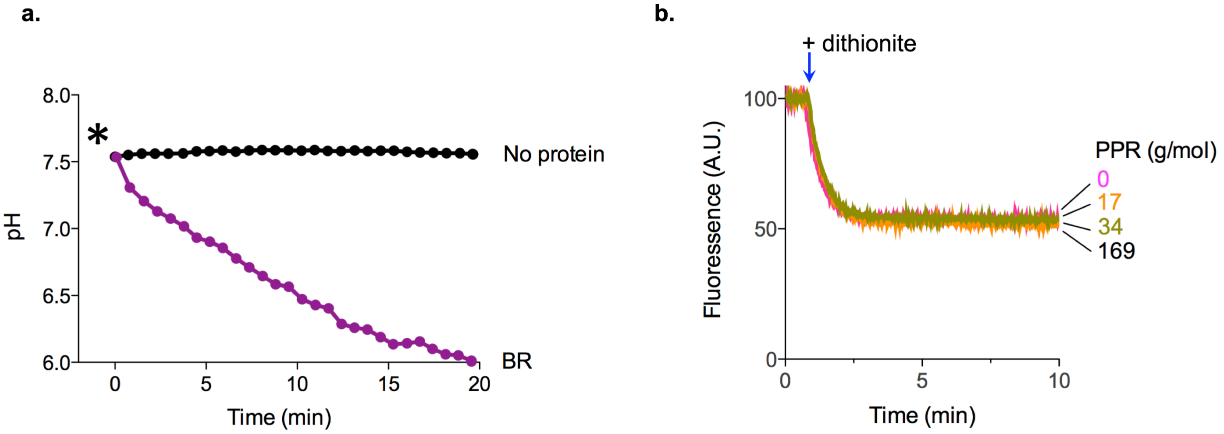

Figure S1: Proton pumping and scramblase activity assays of β-OG-treated BR.

a. Light-induced acidification of the interior of BR-containing proteoliposomes ("BR") measured by the change in the fluorescence of trapped pyranine as described in Fig. 1d-f. Protein-free vesicles ("No protein") were analyzed in parallel. Samples were illuminated (indicated by *) at time = 0 min. **b.** Fluorescence traces from scramblase assays. Vesicles were reconstituted with NBD-PC and different amounts of β-OG-treated BR to generate samples with protein to phospholipid ratio (PPR) values of 17, 34 and 169 g protein/mol phospholipid. A protein-free sample (PPR=0 g/mol) was analyzed in parallel. Assay details are provided in Fig. 2a, b. Note that the samples analyzed here show no scramblase activity despite being reconstituted at much higher PPR values compared with samples containing fully active BR (Fig 2b, c).

Measurement of scramblase activity using back extraction of NBD-phospholipids with fatty acid-free bovine serum albumin (BSA).

We used a 'back-extraction' assay that exploits the observation that the fluorescence of NBD phospholipids is 2-fold lower when the lipids are bound to bovine serum albumin (BSA) compared with when they are located in a liposome membrane[1–3] Thus, the addition of fatty acid-free BSA to large, unilamellar protein-free liposomes is expected to result in a 25% drop in fluorescence (Fig. S2a, upper panel); for a sample where all liposomes contain the BR scramblase, the addition of BSA is expected to result in a 50% drop in fluorescence (Fig. S2a lower panel). As only ~50% of the vesicles contain BR even if high amounts of protein are used for reconstitution (see Results section in the main text), the extent of reduction for proteoliposomes is expected to be 37.5%. We observed ~25% and ~40% reduction in fluorescence for protein-free and BR-containing vesicles, respectively (Fig. S2b), as predicted. Aliquots of the same liposome preparation were also taken for the dithionite-based scramblase assay, showing the expected ~50% and ~75% reduction in fluorescence for protein-free and BR-containing liposomes, respectively (Fig. S2c). Therefore, *the back-extraction assay complements the dithionite-based assay reported in the main text and provides independent verification of BR's phospholipid scramblase activity*.

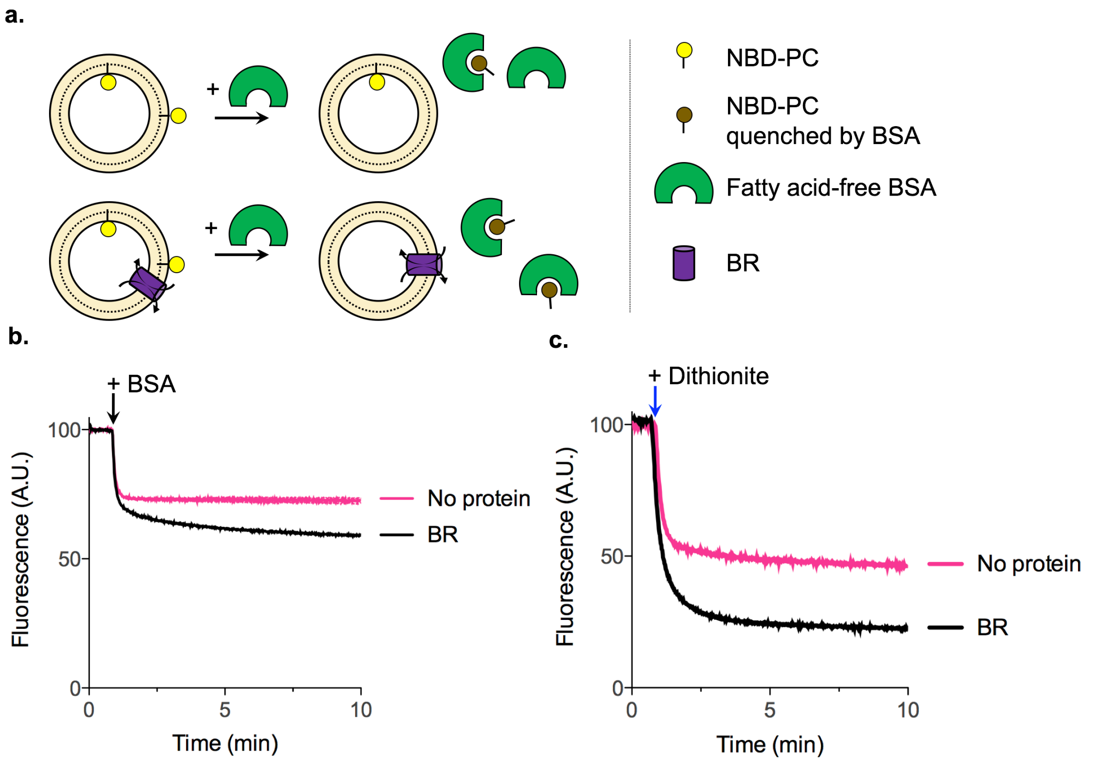

Figure S2: Back extraction assay to measure scramblase activity.

a. Schematic representation of the assay. 1-myristoyl-2-C_6-NBD-PC is used as the fluorescent lipid reporter as the short myristoyl chain enables the lipid to be more readily back-extracted by BSA than the 1-palmitoyl phospholipids typically used for the dithionite-based scramblase assay. The back-extraction assay exploits the fact that the fluorescence of NBD-PC bound to

BSA is ~50% of that of NBD-PC in the membrane. Addition of BSA to protein-free vesicles (top row) extracts NBD-PC in the outer leaflet, resulting in a drop of total fluorescence of the sample by 25%. In the case of BR-containing proteoliposomes, total fluorescence is expected to drop by 50% as all NBD-PC is extracted (NBD-PC molecules originally in the inner leaflet are scrambled to the outer leaflet from where they are extracted by BSA). However, because only 50% of the vesicles are reconstituted with BR even at high protein to phospholipid ratios (see main text), the expected drop in fluorescence is 37.5%. **b.** Scramblase activity of BR revealed by BSA-mediated back extraction of NBD-PC. In protein-free liposomes the addition of fatty acid-free BSA leads to decrease of fluorescence of 25.5 ± 1.7% (mean ± s.d., n=3). In proteoliposomes the decrease of fluorescence is 40.8 ± 0.8% (mean ± s.d., n=3). These values are very close to the expected values of 25 and 37.5%, respectively, for protein-free vesicles and samples reconstituted at a high ratio of BR to phospholipid. **c.** Fresh aliquots of the samples assayed in panel b were assayed for scramblase activity using the dithionite-based method.

Specificity – BR transports common NBD-labeled phospholipids but not M5-DLO.

We tested the ability of BR to scramble various NBD-labeled phospholipids and the isoprenoid-based glycolipid M5-DLO. Fig. S3 shows that NBD-PC, *N*-NBD-PE and C_{12}NBD-SM are all scrambled by BR. Fig. S4 shows that M5-DLO is not a substrate for BR's scramblase activity (as a positive control we reconstituted a crude preparation of yeast endoplasmic reticulum membrane proteins that we previously showed possesses M5-DLO scramblase activity[4,5]). These data indicate that BR is a relatively unspecific phospholipid scramblase; it's inability to scramble M5-DLO could be due to one or more of the structural elements of this lipid (Fig. S4b): large polar headgroup consisting of a heptasaccharide, diphosphate linkage (versus monophosphate in the case of the NBD-phospholipids), long >C70 isoprenoid chain.

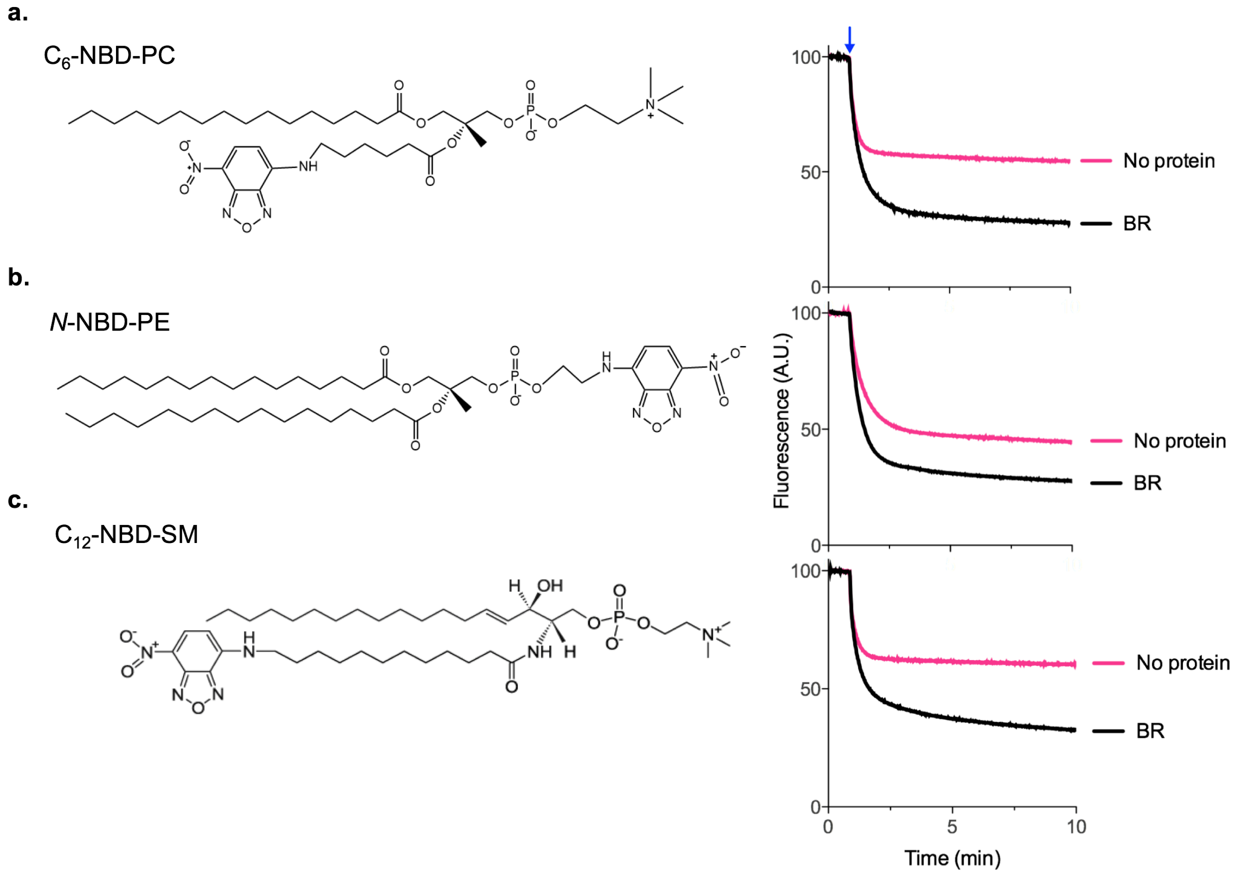

Figure S3: BR scrambles different NBD-labeled phospholipids.

Left panels: abridged version of the name of the fluorescent lipid with its chemical structure shown below. Right panels: fluorescence traces from scramblase assays (carried out as in Fig. 2a, b) comparing protein-free liposomes (pink traces) and BR-containing proteoliposomes (black traces) for different NBD-phospholipids. **a**. C_6-NBD-PC (as in Fig. 2b), **b**. *N*-NBD-PE, **c.** C_{12}-NBD-SM. The arrow in the top right panel indicates the time of dithionite addition for all traces.

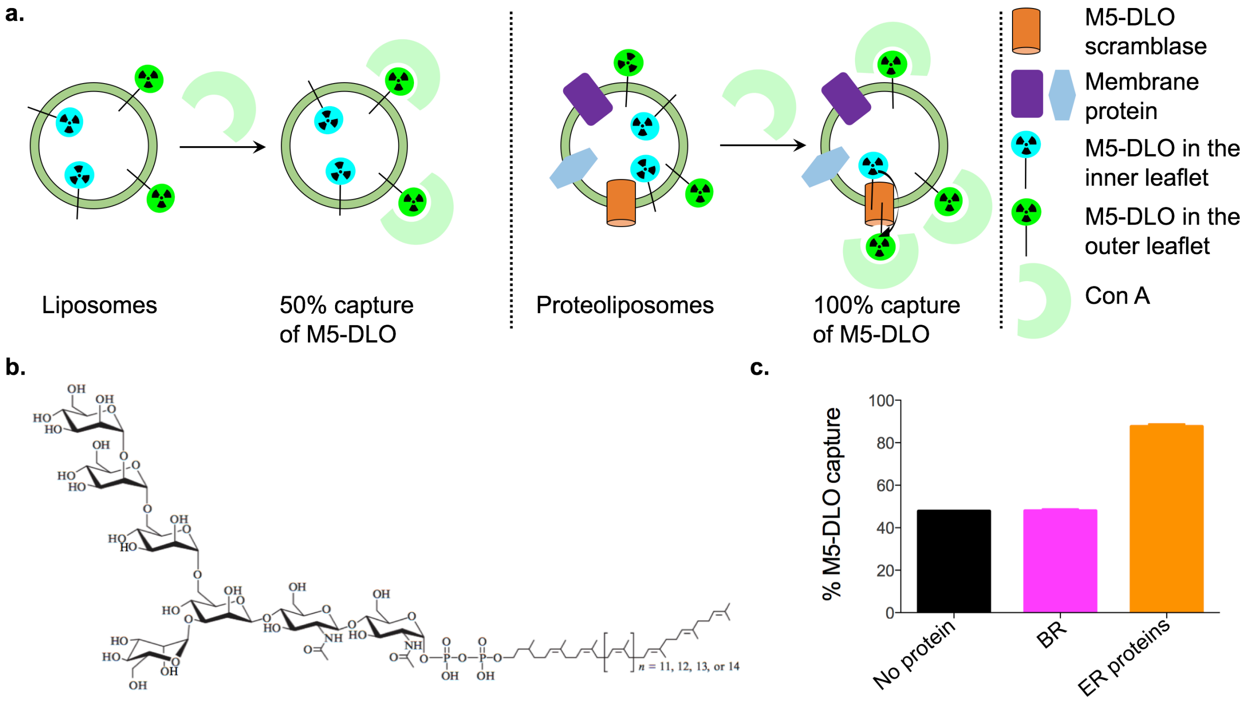

Figure S4: BR does not scramble $Man_5GlcNAc_2$-PP-dolichol (M5-DLO).

a. Schematic representation of the M5-DLO scramblase assay. Liposomes are reconstituted with [^{3}H]M5-DLO and incubated with the mannose-binding lectin Concanavalin A (Con A) to capture M5-DLO molecules in the outer leaflet. Left panel - treatment of protein-free liposomes with Con A results in the capture of 50% of M5-DLO molecules as those in the inner leaflet are protected from Con A; right panel - assay of proteoliposomes containing an M5-DLO scramblase: in this case Con A is expected to capture 100% of M5-DLO molecules as those in the inner leaflet can access the outer leaflet as a result of scramblase activity[4–6]. **b**. Chemical representation of M5-DLO. **c.** Results of the assay comparing control liposomes (No protein), BR proteoliposomes (BR) and proteoliposomes reconstituted with a Triton X-100 extract of yeast endoplasmic reticulum membranes (ER proteins) that contains M5-DLO scramblase activity[4,5]. Values represent means ± range of two independent experiments. DLO, dolichol-linked oligosaccharide; M5-DLO, $Man_5GlcNAc_2$-PP-dolichol; Man, mannose; GlcNAc, *N*-acetylglucosamine, P, phosphate.

Supplementary Information Bibliography

1. Chang, Q., Gummadi, S. N. & Menon, A. K. Chemical modification identifies two populations of glycerophospholipid flippase in rat liver ER. *Biochemistry* **43,** 10710–10718 (2004).
2. Menon, I. *et al.* Opsin Is a Phospholipid Flippase. *Curr. Biol.* **21,** 149–153 (2011).
3. Kubelt, J., Menon, A. K., Müller, P. & Herrmann, A. Transbilayer Movement of Fluorescent Phospholipid Analogues in the Cytoplasmic Membrane of *Escherichia coli*. *Biochemistry* **41,** 5605–5612 (2002).
4. Frank, C. G., Sanyal, S., Rush, J. S., Waechter, C. J. & Menon, A. K. Does Rft1 flip an N-glycan lipid precursor? *Nature* **454,** E3-4-5 (2008).
5. Sanyal, S., Frank, C. G. & Menon, A. K. Distinct Flippases Translocate Glycerophospholipids and Oligosaccharide Diphosphate Dolichols across the Endoplasmic Reticulum. *Biochemistry* **47,** 7937–7946 (2008).
6. Sanyal, S. & Menon, A. K. Specific transbilayer translocation of dolichol-linked oligosaccharides by an endoplasmic reticulum flippase. *Proc. Natl. Acad. Sci.* **106,** 767–772 (2009).
